# *neo*-Clerodane diterpenoids from *Salvia dugesii* and their bioactive studies

**DOI:** 10.1007/s13659-011-0016-6

**Published:** 2011-10-14

**Authors:** Xu Gang, Zhao Fang, Yang Xian-Wen, Zhou Juan, Yang Li-Xin, Xiao-Ling Shen, Ying-Jie Hu, Qin-Shi Zhao

**Affiliations:** 1 State Key Laboratory of Phytochemistry and Plant Resources in West China, Kunming Institute of Botany, Chinese Academy of Sciences, Kunming, 650201 China; 2 Tropical Medicine Institute, Guangzhou University of Chinese Medicine, Guangzhou, 510405 China; 3 Key Laboratory of Marine Bio-resources Sustainable Utilization, South China Sea Institute of Oceanology, Chinese Academy of Sciences, Guangzhou, 510301 China

**Keywords:** *Salvia dugesii*, neo-clerodane diterpenoids, dugesins, antivirus

## Abstract

*Salvia dugesii* is an invasive plant in Yunnan, China. To tentatively explore its utilization, a systematic phytochemical investigation was carried out on this plant, which led to the isolation of five new *neo*-clerodane diterpenoids, dugesins C–G (**1**–**5**), together with six known ones. Their structures were determined by comprehensive NMR and MS spectroscopic analysis. It was noteworthy that the eleven isolates, composed of five different carbocyclic systems derived from the *neo*-clerodane diterpenoid skeleton, were reported from the same plant for the first time. The anti-feedantial, cytotoxic, and antiviral activities of the isolates were evaluated. Dugesin F (**4**) was tested to be a non-toxic antiviral compound against influenza virus FM1.

**Electronic Supplementary Material:**

Supplementary material is available for this article at 10.1007/s13659-011-0016-6 and is accessible for authorized users.

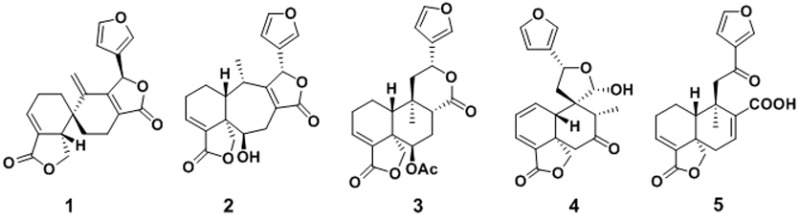

## Introduction

The problem caused by invasive species has become increasingly important in recent years. *Salvia dugesii* Fernald is native to Mexico and was introduced to Yunnan province of China in late 1980s. Its rapid spread in the ecosystem as an invasive plant and has caused increasing threats to the local agricultural and horticultural systems.[1] Clerodane diterpenoids, a type of characteristic secondary metabolites of *Salvia* species, have been reported frequently with diverse biological activities from different *Salvia* species distributed in Mexico and Central and South America.[2–7] Previously chemical studied on the title plant afforded several *neo*-clerodane diterpenoids.[8] With the aim of searching for useful metabolites from this invasive plant and continuing our systematic studies of *Salvia* species,[8–10] the chemical constituents of *S. dugesii* was investigated, and five new *neo*-clerodane diterpenoids (dugesins C–G, **1**–**5**) were isolated together with six known ones: dugesins A and B,[8] tilifodiolide,[3] isosalvipuberulin,[4] salviandulin E,[5] and salvifaricin.[11,12] In order to assess the potential usage of these isolates, their cytotoxic, antifeedantial, and antiviral activities were evaluated. Dugesin F (**4**) was shown to be a non-toxic antiviral compound against influenza virus FM1. Reported herein were the isolation, structure, and bioactivity evaluation of these metabolites.

*To whom correspondence should be addressed. E-mail: xugang008@mail.kib.ac.cn; xlshen66@hotmail.com

## Results and Discussion

Springer

The acetone extract of the aerial parts of *S. dugesii* was repeatedly chromatographed over silica gel, Lichroprep RP-18, and Sephadex LH-20 to afford five new neoclerodane diterpenoids (dugesins C–G, **1**–**5**), and six known ones, dugesins A and B, tilifodiolide, isosalvipuberulin, salviandulin E, and salvifaricin. It was interesting that the eleven isolates, composed of five different carbocyclic systems derived from the normal *neo*-clerodane diterpenoid skeleton, were reported from the same plant for the first time. It was also noteworthy that the skeleton of dugesins A and D, and salviandulin E, possessing a 6/7 carbocyclic systems derived from the normal *neo*-clerodane skeleton, respectively, was very rare in nature.[5,6,8,13,14] Dugesin C, possessing a spirocyclic carbocyclic system derived from the normal *neo*-clerodane skeleton, was the third example of natural metabolites with such a novel carbocyclic skeleton.[15] The major constituent (content about 0.2% in the dry plant sample) of *S. dugesii*, tilifodiolide, can be seen as the only tetraline-type diterpenoid of clerodanic origin in nature products up to now.[3] In order to assess the potential usage of these isolates, their antifeedantial, cytotoxic, anti-malarial, and antiviral activities were evaluated, and dugesin F (**4**) was tested to be a non-toxic antiviral compound against influenza virus FM1. It was noteworthy that dugesins C and D, possessing a 6/7 carbocyclic system derived from the normal *neo*-clerodane skeleton, were two salvigenane diterpenoids which were very rare in nature.

Dugesin C (**1**) was obtained as white amorphous powder, giving a molecular ion peak at *m/z* 338 in the EIMS spectrum. It was deduced to have a molecular formula $C_{20}H_{18}O_5$ by HRESIMS and the $^{13}C$ NMR spectrum (Table 1). The IR spectrum showed the presence of furan ring (1504 and 874 $cm^{-1}$) and $\alpha,\beta$-unsaturated $\gamma$-lactone moieties (1754 and 1682$cm^{-1}$).[2–8] The $^{13}C$ NMR and DEPT spectra of **1** exhibited signals for 20 carbons, including two lactonic carbonyls, six quaternary carbons, six methines (including four unsaturated ones and an oxygenated one), and six methylenes (one unsaturated one and one oxygenated). Considering many *neo*-clerodane diterpenoids have been isolated from different *Salvia* species previously, along with the spectral features obseverd in the NMR spectra, **1** could be ascribed to be a *neo*-clerodane diterpenoid.[3–8,15] Carefully analysis of the NMR spectral data indicated that the 1D NMR data (Table 1) of **1** are very similar to those of salvileucalin A, a novel spirocyclic diterpenoid with a rearranged *neo*-clerodane keleton from *Salvia leucantha*.[15] The differences observed in the $^{1}H$ and $^{13}C$ NMR spectra were the presence of one methylene ($\delta_C$ 23.5, C-2) and a methine ($\delta_C$ 44.6, C-5) signals in **1** instead of the signals for the unsaturated methine at C-2 and the unsaturated quaternary carbon at C-5 in salvileucalin A. These differences suggested that the two double bonds of $\Delta^{2(3)}$ and $\Delta^{4(5)}$ in salvileucalin A was replaced by $\Delta^{3(4)}$ in **1**, which can be confirmed by the HMBC correlations from H-20 ($\delta_H$ 5.16 and 5.10, each 1H) to C-1, C-9, C-10, and C-11, from H-3 ($\delta_H$ 6.73, s) to C-1, C-2, C-5, and C-18, and from H-5 ($\delta_H$ 3.16, m) to C-1, C-4, C-6, C-9, C-10, and C-19. In addition, two key correlation systems of H-1/H-2/H-3 and H-5/H-19 in the $^{1}H$-$^{1}H$ COSY spectrum was evident in **1**, which also confirm its major difference between salvileucalin A.

The relative configuration of **1** was elucidated to be the same with that of salvileucalin A by analysis of ROESY spectrum (Figure 1). The ROESY cross-peaks of H-5/H-6$\beta$, H-5/H-1$\beta$, H-19/H-20, and H-19/H-6$\alpha$, established the $\beta$-orientation of H-5 and C-6. H-12 was deduced to be $\alpha$-oriented by its NOE correlation with H-20. On the basis of all the above evidence, the structure of **1** was established and named dugesin C.

**Table 1.** $^{13}C$ **NMR spectroscopic data for compounds 1-5 (100 MHz;** $\delta$ **in ppm).**

| Pos. | **1**[a] | **2**[b] | **3**[a] | **4**[c] | **5**[a] |
|---|---|---|---|---|---|
| 1 | 30.0 $CH_2$ | 28.3 $CH_2$ | 19.9 $CH_2$ | 126.2 CH | 20.1 $CH_2$ |
| 2 | 23.5 $CH_2$ | 26.1 $CH_2$ | 27.2 $CH_2$ | 128.7 CH | 26.7 $CH_2$ |
| 3 | 136.3 CH | 138.9 CH | 138.9 CH | 137.1 CH | 136.8 CH |
| 4 | 127.3 C | 137.2 C | 134.7 C | 132.0 C | 136.0 C |
| 5 | 38.5 C | 54.9 C | 49.4 C | 60.5 C | 43.7 C |
| 6 | 35.1 $CH_2$ | 67.4 CH | 68.8 CH | 44.3 $CH_2$ | 36.4 $CH_2$ |
| 7 | 17.2 $CH_2$ | 29.5 $CH_2$ | 24.0 $CH_2$ | 208.4 C | 141.4 CH |
| 8 | 126.7 C | 123.8 C | 42.9 CH | 49.4 CH | 135.8 C |
| 9 | 135.9 C | 38.9 CH | 36.6 C | 60.5 C | 40.0 C |
| 10 | 44.6 CH | 35.7 CH | 46.6 CH | 46.3 CH | 44.8 CH |
| 11 | 156.4 C | 167.6 C | 43.4 $CH_2$ | 43.8 $CH_2$ | 46.0 $CH_2$ |
| 12 | 74.3 CH | 77.3 CH | 70.0 CH | 74.5 CH | 193.9 C |
| 13 | 121.1 C | 121.3 C | 124.3 C | 130.5 C | 128.6 C |
| 14 | 107.6 CH | 109.1 CH | 108.4 CH | 110.5 CH | 108.5 CH |
| 15 | 144.6 CH | 144.9 CH | 144.0 CH | 144.2 CH | 147.4 CH |
| 16 | 141.8 CH | 142.7 CH | 139.6 CH | 140.7 CH | 144.3 CH |
| 17 | 171.9 C | 174.8 C | 172.8 C | 9.7 $CH_3$ | 170.3 C |
| 18 | 169.3 C | 169.4 C | 167.1 C | 168.6 C | 168.9 C |
| 19 | 67.0 $CH_2$ | 71.1 $CH_2$ | 70.8 $CH_2$ | 76.5 $CH_2$ | 72.4 $CH_2$ |
| 20 | 118.5 $CH_2$ | 15.1 $CH_3$ | 19.0 $CH_3$ | 98.9 CH | 22.0 $CH_3$ |
| $CH_3$*CO* | | | 169.3 C | | |
| *$CH_3$*CO | | | 20.9 $CH_3$ | | |

[a]recorded in $CDCl_3$; [b]recorded in $C_5D_5N$; [c]recorded in acetone-$d_6$.

**Figure 1.** Key ROESY correlations for dugesin C (**1**).

The HRESIMS indicated that **2** (dugesin D) has a molecular formula of $C_{20}H_{20}O_6$. Spectroscopic data interpretation indicated that the structure of **2** was also closely similar to dugesin A.[8] The differences in the 1D NMR spectral data of **2** compared to dugesin A were the presence of three more methines (including a oxygenated one) instead of two olefinic carbons (C-9 and C-10) and a methylene signal (C-6) in dugesin A, which indicated C-9 and C-10 were two saturated methines and C-6 was substituted by a hydroxyl group. This deduction can be confirmed by HMBC correlations observed from H-6 to C-4, C-5, C-8, and C-10, from H-10 to C-1, C-5, C-9, C-19, and C-20, and from H-9 to C-1, C-5, C-8, C-10, C-11, C-12, and C-20. The relative configuration of **2** was established on the basis of ROESY experiment. The NOE correlations of H-19 with Me-20 and H-6 indicated the $\alpha$-orientation of both H-6 and Me-20. In addition, the NOE correlations for H-9/H-10 and H-9/H-12 indicated that H-10 and H-12 were both $\beta$-oriented. So, the structure of **2** can be described as 6$\beta$-hydroxy-3,8-salvigenadiene, and named dugesin D.

The molecular formula of dugesin E (**3**) was determined to be $C_{22}H_{24}O_7$ from its HRESIMS and NMR spectral data. The $^{13}C$ NMR spectrum (Table 1) exhibited signals for seven quaternary carbons, eight methines, five methylenes, and two methyls. A side-by-side comparison of the 1D NMR spectral data of **3** and bacchotricuneatin A showed that **3** was the acetoxyl derivative of bacchotricuneatin A.[16] The acetoxyl group was located at C-6 by the HMBC correlations observed between H-6 ($\delta_H$ 5.33, s) with C-4, C-5, C-8, C-19, and the acetoxyl carbonyl at $\delta_C$ 169.3 (s). The relative configuration of **3** was suggested to be the same as that of bacchotricuneatin A by the analysis of the ROESY correlations (Figure 2). The NOE correlation between H-6 and H-19 indicated the $\alpha$-orientation of H-6. In the same spectrum, the correlations for H-8/H-12, H-8/H-10, Me-20/H-19, and H-1$\alpha$/H-19 confirmed H-8, H-10, and H-12 were all $\beta$-oriented while Me-20 was in the $\alpha$-orientation. Thus, the structure of **3** was elucidated and the trivial name was proposed as dugesin E.

**Figure 2.** Key ROESY correlations for dugesin E (**3**).

Dugesin F (**4**) has the molecular formula $C_{20}H_{20}O_6$ deduced from the HRESIMS. The NMR spectral data of **4** were closely comparable to those of salvifolin except for the lack of an acetyl group at C-20,[3] which indicated that compound **4** was the 20-deacetyl derivative of salvifolin. This deduction can be confirmed by the HMBC correlations from H-20 to C-8, C-9, C-10, C-11, and C-12. The relative configuration of **4** was indicated to be the same as that of salvifolin by the comparative analysis of the ROESY spectrum (Figure 3). The NOE correlations for H-10/H-6$\beta$, H-10/H-8, H-6$\beta$/H-8, and H-20/H-19 observed in the ROESY spectrum indicated the $\beta$-orientation of H-8, H-10, and H-20. The configuration of H-12 was $\alpha$-oriented as indicated by the NOE correlation of Me-17/H-12. On this basis, the structure of **4** was determined and named dugesin F.

**Figure 3.** Key ROESY correlations for dugesin F (**4**).

The molecular formula $C_{20}H_{20}O_6$ was determined for dugesin G (**5**) by the HRESIMS. The $^1H$ and $^{13}C$ NMR signals for one tertiary methyl, a $\beta$-substituted furan ring, an carboxyl, and an $\alpha,\beta$-unsaturated $\gamma$-lactone moieties were evident. Carefully spectroscopic data interpretation indicated that the structure of **5** was very similar to nasimalun A, a *neo*-clerodane diterpenoid previously isolated from *Barringtonia racemosa*.[17]

**Table 2.** $^1H$ **NMR spectroscopic data for 1-5 (400 MHz; $\delta$ in ppm, $J$ in Hz).**

| Pos. | **1**[a] | **2**[b] | **3**[a] | **4**[c] | **5**[a] |
|---|---|---|---|---|---|
| 1$\alpha$ | 1.88 m | 1.35 m | 1.22 m | 6.40 m | 1.23 m |
| 1$\beta$ | 1.53 m | | 1.76 m | | 1.84 m |
| 2a | 2.40 m | 2.13 m | 2.40 m | 6.50 m | 2.40 m |
| 2b | 1.90 m | | 2.21 m | | 2.11 m |
| 3 | 6.73 s | 7.07 s | 6.84 d (6.1) | 6.97 d (5.0) | 6.83 d (6.0) |
| 5 | 3.16 m | | | | |
| 6$\alpha$ | 1.89 m | 4.45 d (3.5) | 5.33 s | 2.07 m | 2.55 dd (6.3, 18.3) |
| 6$\beta$ | 1.70 m | | | 2.62 m | 2.19 d (18.3) |
| 7a | 2.50 m | 3.40 dd (3.5, 17.4) | 2.21 m | | 7.22 d (6.3) |
| 7b | | 3.13 d (17.4) | | | |
| 8 | | | 2.96 dd (6.5, 9.8) | 3.04 m | |
| 9 | | 2.67 dd (7.3, 9.2) | | | |
| 10 | 3.16 m | 3.31 m | 2.39 m | 3.26 m | 2.61 m |
| 11a | | | 2.20 m | 2.83 dd (8.1, 13.2) | 3.72 d (16.3) |
| 11b | | | 1.93 m | 2.21 dd (8.1, 13.2) | 2.61 d (16.3) |
| 12 | 6.01 s | 6.01 s | 5.44 m | 5.18 t (8.1) | |
| 14 | 6.09 s | 6.44 s | 6.41 s | 6.54 s | 6.66 s |
| 15 | 7.36 s | 7.66 s | 7.42 s | 7.44 s | 7.98 s |
| 16 | 7.51 s | 7.92 s | 7.46 s | 7.50 s | 7.36 s |
| 17 | | | | 1.13 d (7.0) | |
| 19a | 4.29 t (9.2) | 4.31 d (8.8) | 4.15 d (8.6) | 4.13 d (9.1) | 3.99 s |
| 19b | 4.51 t (9.2) | 3.13 d (8.8) | 4.01 d (8.6) | 4.47 d (9.1) | |
| 20a | 5.16 s | 0.72 d (7.3) | 0.83 s | 5.16 s | 1.12 s |
| 20b | 5.10 s | | | | |
| *$CH_3$*CO | | | 2.02 s | | |

[a]recorded in $CDCl_3$; [b]recorded in $C_5D_5N$; [c]recorded in acetone-$d_6$.

It was evident that signals for the methoxyl group in nasimalun A was absent in the 1D NMR spectra of **5**. The other notable difference was that the oxygenated methine (C-8) and a methylene (C-7) in nasimalun A were changed into a double bond in **5**. This was supported by HMBC correlations from H-7 to C-5, C-6, C-8, C-9, and C-17. In the ROESY spectrum, the correlations of Me-20/H-19, H-19/H-1$\alpha$, H-1$\beta$/H-10 can be found, which indicated Me-20 was in the $\alpha$-orientation while H-10 was $\beta$-oriented. Then, the structure of **5** (dugesin G) was thus established as depicted.

To explore the usage of this invasive plant, metabolites of this plant had been assessed in human tumor cell lines as well as in influenza virus FM1 strain. Firstly, the eleven isolates were evaluated for cytotoxicity toward human tumor cells HepG2, CNE, Hela, and NCI-H460, but none of them exhibited promising antitumor activity with $IC_{50} < 20$ $\mu$M. In antiviral assay, sample were tested *in vitro* anti-influenza ability by Cytopathic effect (CPE) and hemagglutination (HI) test. Under their non-toxic concentrations to dog kidney MDCK cells (non-toxic concentration of **4** to cells is 9.78 uM). dugesin F (**4**) was the only one exhibiting inhibitory effect on influenza virus FM1 strain causing CPE in MDCK cells. This isolates at 31.25 $\mu$g/mL completely inhibited influenza virus FM1 strain in HI test. $TC_{50}$ value and $IC_{50}$ value of this compound determined by MTT assay were 45.67 $\mu$g/mL and 9.43 $\mu$g/mL, respectively. Corresponding therapeutic index (TI) was 4.84, implying dugesin F (**4**) a non-toxic antiviral compound.

Considering the fact that clerodane diterpenoids have reported as a rich source of natural insect antifeedants,[18] the antifeedant activity of the four major constituents tilifodiolide, compound **1**, isosalvipuberulin, and salviandulin E was also evaluated. The result indicated that dugesin E and isosalvipuberulin exhibited weak anti-feedant activities with AI (antifeedant index, %) of 6.49 and 14.94, respectively. Tilifodiolide and salviandulin E had no anti-feedant activity at all. Instead, they can improve the Pseudaletia separata Walker to eat fresh leaves of Vicia faba L., especially for tilifodiolide (Table 3).

## Experimental Section

**General Experimental Procedures**. Optical rotations were measured with a Horiba SEPA-300 polarimeter. Ultraviolet absorption spectra were recorded on a UV-2401 PC spectrophotometer. IR spectra were obtained from a Bio-Rad FtS-135 spectrometer. NMR spectra were measured on a Bruker AV-400 spectrometer with TMS as the internal standard, while 2D NMR spectra were recorded by using a Bruker DRX-500 NMR instrument. EIMS were obtained on a VG Auto Spec-3000 spectrometer (70 eV). ESIMS and HRESIMS were taken on an API Qstar Pulsar instrument. Column chromatography was performed on silica gel and silica gel H (200–300 mesh, 10–40 $\mu$m, Qingdao Marine Chemical Inc. China). Lichroprep RP-18 (43–63 $\mu$m, Merck, Darmstadt, Germany), Sephadex LH-20 (Pharmacia Fine Chemical Co. Ltd.), and DM-130 (macroporous resin, Baoen Co. Ltd.) were also used for column chromatography. An Agilent 1100 series instrument equipped with Alltima $C_{18}$ column (4.6 × 250 mm) was used for HPLC analysis and a preparative Alltima $C_{18}$ column (22 × 250 mm) was used in the sample preparation. Fractions were monitored by TLC and spots were visualized by heating silica gel plates sprayed with 10% $H_2SO_4$ in EtOH.

**Plant Material.** Leaves and stems of *S. dugesii* Fernald were collected in Kunming, Yunnan, China, in August, 2006, and were identified by Prof. Xi-Wen Li of the Kunming institute of Botany, the Chinese Academy of Sciences, China. A voucher specimen, 2006218, was deposited in the State Key Laboratory of Phytochemistry and Plant Resources in West China, Kunming Institute of Botany, Chinese Academy of Sciences.

**Extraction and Isolation.** The dried and powdered (8.8 kg) *S. dugesii* Fernald were extracted with $Me_2CO$ for three times (4 × 40 L, each 3 days) at room temperature and filtered. The solvent was removed under vacuum. The gummy residue (310 g) was subjected to column chromatography over DM-130 porous resin and eluted with MeOH-$H_2O$ (50% and 90%). The residue of 90% MeOH-$H_2O$ fraction was partitioned between $H_2O$ and EtOAc. The EtOAc part (210 g) was subjected to column chromatography over silica gel. Mixtures of petroleum ether-$Me_2CO$ of increasing polarity were used as eluents. Five fractions were collected and combined by monitoring with TLC. Tilifodiolide (27.5 g) was crystallized from the second fraction. The mother liquor of the second fraction was chromatographed on a silica gel column (petroleum ether-$CHCl_3$-EtOAc, 4:4:2) and sephadex LH-20 ($CH_3Cl$-MeOH, 1:1) to give compound **1** (78 mg), dugesin A (8 mg), and isosalvipuberulin (645 mg). Fraction 3 was repeatedly chromatographed over lichroprep RP-18 eluted by MeOH-$H_2O$ (from 5:5 to 1:0) and silica gel (petroleum ether-$Me_2CO$, 94:6) to give compound **3** (10 mg) and salvifaricin (9 mg). Fraction 4 was chromatographed on lichroprep RP-18 (MeOH-$H_2O$, from 65:35 to 100:0) to afford three sub-fractions, 3a–3c. Sub-fraction 3a was subjected to column chromatography on silica gel using petroleum ether-$Me_2CO$ (9:1) as eluent to yield the crude compounds **2** (8 mg), **4** (6 mg), dugesin B (4 mg), and salviandulin E (18 mg), which was purified by sephadex LH-20 ($CH_3Cl$-MeOH, 1:1) and preparative HPLC (MeOH-$H_2O$, 9:1). Sub-fraction 3b was further purified by Sephadex LH-20 ($CH_3Cl$-MeOH, 1:1) and preparative HPLC (MeOH-$H_2O$, 85:15) to afford compound **5** (2 mg).

**Dugesin C (1):** white powder; $[\alpha]_D^{22}$ − 32.4 (*c* 0.40, $CHCl_3$).

**Table 3. Antifeedant activities of the isolates with AI (antifeedant index, %) at 500 ppm.**

| compound | feeding area (mm$^2$) | | | | | | | | | | average feeding area (mm$^2$) | AI |
|---|---|---|---|---|---|---|---|---|---|---|---|---|
| | 1 | 2 | 3 | 4 | 5 | 6 | 7 | 8 | 9 | 10 | | |
| dugesin E | 144 | 290 | 302 | 330 | 289 | 212 | 311 | 336 | 257 | 207 | 267.8 | 6.49 |
| isosalvipuberulin | 234 | 252 | 263 | 273 | 279 | 225 | 249 | 301 | 262 | 98 | 243.6 | 14.94 |
| tilifodiolide | 356 | 353 | 348 | 339 | 343 | 357 | 370 | 355 | 323 | 376 | 352 | − 22.91 |
| salviandulin E | 260 | 293 | 243 | 293 | 308 | 358 | 348 | 366 | 339 | 312 | 312 | − 8.94 |
| CK | 215 | 295 | 245 | 281 | 325 | 296 | 313 | 286 | 270 | 338 | 286.4 | 0 |

IR (KBr) $\upsilon_{max}$ 2923, 1754, 1682, 1657, 1504, 1432, 1420, 1207, 1190, 1019, 957, 874, 752 $cm^{-1}$. UV ($CHCl_3$) $\lambda_{max}$ (log ε) 256.2 (1.54) nm. $^1H$ ($CDCl_3$, 400MHz) and $^{13}C$ NMR ($CDCl_3$, 100MHz) spectral data, see Tables 1 and 2. EIMS *m/z* 338 $[M]^+$ (12), 320 (14), 292 (20), 270 (41), 220 (100), 228 (45), 195 (59), 161 (100), 153 (44) , 141 (56), 128 (84), 115 (73), 95 (69). Positive HRESIMS *m/z* 339.1230 $[M + H]^+$, (calcd. for $C_{20}H_{19}O_5$, 339.1233).

**Dugesin D (2):** white powder; $[\alpha]_D^{22} - 228.5$ (*c* 0.43, $CHCl_3$). IR (KBr) $\upsilon_{max}$ 3436, 2927, 1755, 1675, 1504, 1432, 1304, 1262, 1192, 1164, 1026, 994, 875 $cm^{-1}$. UV ($CHCl_3$) $\lambda_{max}$ (log ε) 256.2 (1.08), 239.4 (1.19) nm. $^1H$ ($C_5D_5N$, 400Hz) and $^{13}C$ NMR ($C_5D_5N$, 100Hz) spectral data, see Tables 1 and 2. EIMS *m/z* 356 $[M]^+$ (9), 338 (9), 326 (76), 308 (14), 280 (16), 220 (100), 191 (72), 176 (26), 145 (24), 95 (40). Positive HRESIMS *m/z* 379.1153 $[M + Na]^+$, (calcd. for $C_{20}H_{20}O_6Na$, 379.1157).

**Dugesin E (3):** white powder; $[\alpha]_D^{22} - 41.0$ (*c* 0.41, $CHCl_3$). IR (KBr) $\upsilon_{max}$ 2956, 1768, 1662, 1504, 1432, 1375, 1288, 1237, 1196, 1147, 1024, 873 $cm^{-1}$. UV ($CHCl_3$) $\lambda_{max}$ (log ε) 268.8 (0.44), 239.6 (0.65) nm. $^1H$ ($CDCl_3$, 400Hz) and $^{13}C$ NMR ($CDCl_3$, 100Hz) spectral data, see Tables 1 and 2. EIMS *m/z* 400 $[M]^+$ (16), 358 (6), 340 (6), 256 (24), 219 (36), 203 (86), 135 (63), 95 (81), 82 (100). Positive HRESIMS *m/z* 423.1420 $[M + Na]^+$, (calcd. for $C_{22}H_{24}O_7Na$, 423.1419).

**Dugesin F (4):** white powder; $[\alpha]_D^{22} - 26.1$ (*c* 0.08, $CHCl_3$). IR (KBr) $\upsilon_{max}$ 3432, 2923, 1754, 1712, 1631, 1503, 1458, 1341, 1261, 1229, 1038, 1008, 969, 874 $cm^{-1}$. UV ($CHCl_3$) $\lambda_{max}$ (log ε) 275.4 (1.45), 239.0 (1.39) nm. $^1H$ ($CDCl_3$, 400Hz) and $^{13}C$ NMR (acetond-$d_6$, 100Hz) spectral data, see Tables 1 and 2. EIMS: *m/z* 356 $[M]^+$ (3), 338 (11), 267 (12), 239 (18), 203 (12), 185 (13), 165 (13), 134 (22), 128 (20), 115 (28), 108 (87), 95 (49), 94 (100), 81 (43). Positive HRESIMS *m/z* 379.1164 $[M + Na]^+$, (calcd. for $C_{20}H_{20}O_6Na$, 379.1157).

**Dugesin G (5):** white powder; $[\alpha]_D^{22} - 88.9$ (*c* 0.02, $CHCl_3$). IR (KBr) $\upsilon_{max}$ 3455, 2916, 1769, 1725, 1670, 1504, 1557, 1364, 1268, 1229, 1039, 1018, 979, 874 $cm^{-1}$. UV ($CHCl_3$) $\lambda_{max}$ (log ε) 240.8 (0.96), 230.0 (0.80) nm. $^1H$ ($CDCl_3$, 400MHz) and $^{13}C$ NMR ($CDCl_3$, 100MHz) spectral data, see Tables 1 and 2. EIMS: *m/z* 356 ($M^+$, 2), 338 (13), 310 (9), 246 (15), 216 (11), 201 (25), 171 (12), 157 (20), 143 (34), 128 (19), 110 (100), 95 (45). Positive HRESIMS *m/z* 379.1165 $[M + Na]^+$, (calcd. for $C_{20}H_{20}O_6Na$, 379.1157).

**Anti-Viral Bioassay.** Anti-viral activity was tested using cytopathic effect (CPE) reduction assay and hemagglutination (HI) in influenza virus FM1 strain infected dog kidney cell line MDCK by referring method described previously.[19] Briefly, virus infected MDCK cells were treated with different concentrations of sample. A known antiviral agent virazole was used as positive control. Drug treatment lasted for 4 d. The CPE was examined and counted and the concentration that reduced CPE by 50% was recorded as $IC_{50}$. The media were checked by HI test for virus caused blood clotting. Virus uninfected MDCK cells were treated the same for determination of $TC_{50}$ (concentration of sample causing 50% of cell death). Therapeutic index (TI) of sample is calculated from the ratio of $TC_{50}/IC_{50}$. A drug that has a TI > 2 is thought to be an effective virus inhibitor and be non-toxic. While in HI test, an effective virus inhibitor should inhibit the formation of blood clot.

**Cytotoxic Bioassay.** The cytotoxicity of selected isolates against HepG2, CNE, Hela and NCI-H460 cell lines was investigated using MTT assay.[20] Concentration of a compound inhibiting 50% of cell growth ($IC_{50}$) of each compound was calculated by the Reed and Muench method. Solvent and blank were included in experiments.[21]

**Antifeedant Bioassay.** The tested samples were dissolved in acetone and diluted to 500 ppm using 0.1% Triton X-100 in water. The fresh leaves of *Vicia faba L.* were cut into small holes of 189 $mm^2$ area by a puncher. The cotyledon leaves were dipped into different sample solutions for 3 seconds, and air dried. Solvent control (0.1% Triton X-100 in water) was used as an untreated group. The antifeedant experiments were then carried out by a modified leaf-disk choice test.[22] After the third-instar *Pseudaletia separate*, Walker was starved for 3 h, it was put into a Petri dish with two treated and untreated cotyledon leaves and allowed to feed for 24 h. Ten replicates were used for each treatment.

## Electronic Supplementary Material

Supplementary material is available in the online version of this article at http://dx.doi.org/10.1007/s13659-011-0016-6 and is accessible for authorized users.

## Acknowledgments

This work was supported financially by the National Basic Research Program of China (2009CB522300), the National Natural Science Foundation of China (No. 20702054), Shanghai Landscaping Administration Bureau Program (G102404), the foundation from the Chinese Academy of Sciences (2010KIBA10), and the Young Academic and Technical Leader Raising Foundation of Yunnan Province (No. 2009CI073).

**Open Access** This article is distributed under the terms of the Creative Commons Attribution License which permits any use, distribution, and reproduction in any medium, provided the original author(s) and source are credited.

## Electronic supplementary material

Supplementary material, approximately 815 KB.
