# Supplementary material for: neo-Clerodane diterpenoids from Salvia dugesii and their bioactive studies
Source: Nat Prod Bioprospect. 2011 Oct 14;1(2):81–6. doi: 10.1007/s13659-011-0016-6 (PMC4131648; doi:10.1007/s13659-011-0016-6)
Supplement: Supplementary file 1 — Supplementary material, approximately 815 KB. [file 13659_2011_16_MOESM1_ESM.pdf]

Electronic Supplementary Material

neo-Clerodane diterpenoids from *Salvia dugesii* and their bioactive studies

Gang XU,[a,*] Fang ZHAO,[b] Xian-Wen YANG,[c] Juan ZHOU,[b] Li-Xin YANG,[a] Xiao-Ling SHEN,[b,*] Ying-Jie HU,[b] and Qin-Shi ZHAO[a]

[a]State Key Laboratory of Phytochemistry and Plant Resources in West China, Kunming Institute of Botany, Chinese Academy of Sciences, Kunming 650201, China

[b]Tropical Medicine Institute, Guangzhou University of Chinese Medicine, Guangzhou 510405, China

[c]Key Laboratory of Marine Bio-resources Sustainable Utilization, South China Sea Institute of Oceanology, Chinese Academy of Sciences, Guangzhou 510301, China

Received 7 September 2011; Accepted 21 September 2011

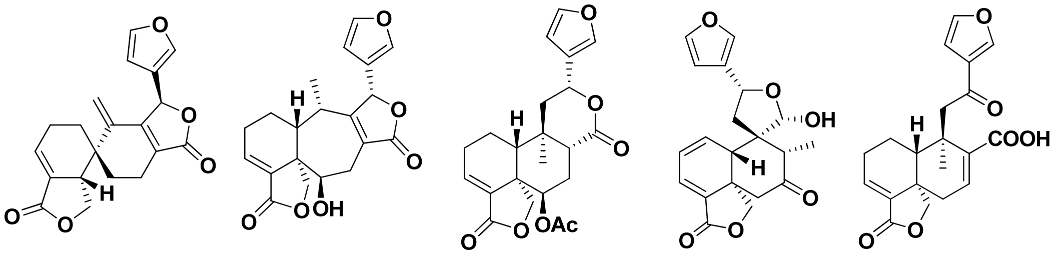

1 2 3 4 5

Structures of compounds **1**–**5**.

*To whom correspondence should be addressed. E-mail: xdluo@mail.kib.ac.cn.

Electronic Supplementary Material List
Characterization Data of New Compounds

► **Compound 1：1D, 2D NMR and HRESIMS**

► **Compound 2：^{1}H and ^{13}C NMR**

► **Compound 3：^{1}H and ^{13}C NMR**

► **Compound 4：^{1}H and ^{13}C NMR**

► **Compound 5：^{1}H and ^{13}C NMR**

For compounds 1:

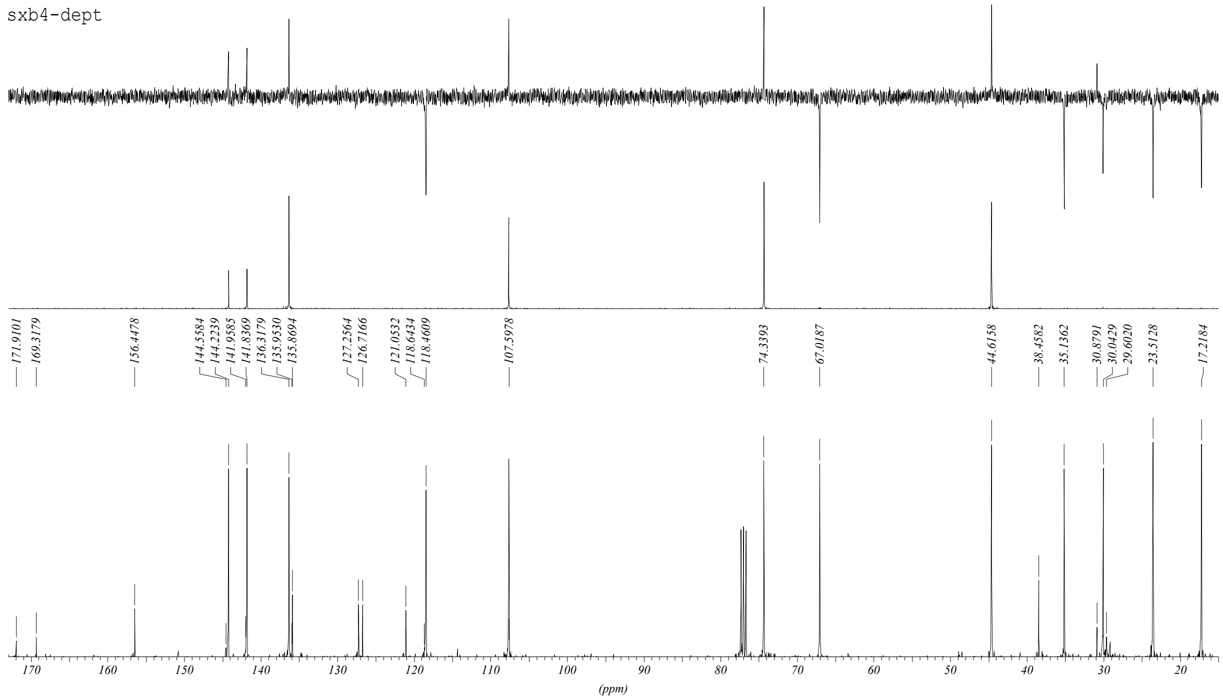

SSpe

ctra1. ^{13}C NMR and DEPT spectra of compound **1**

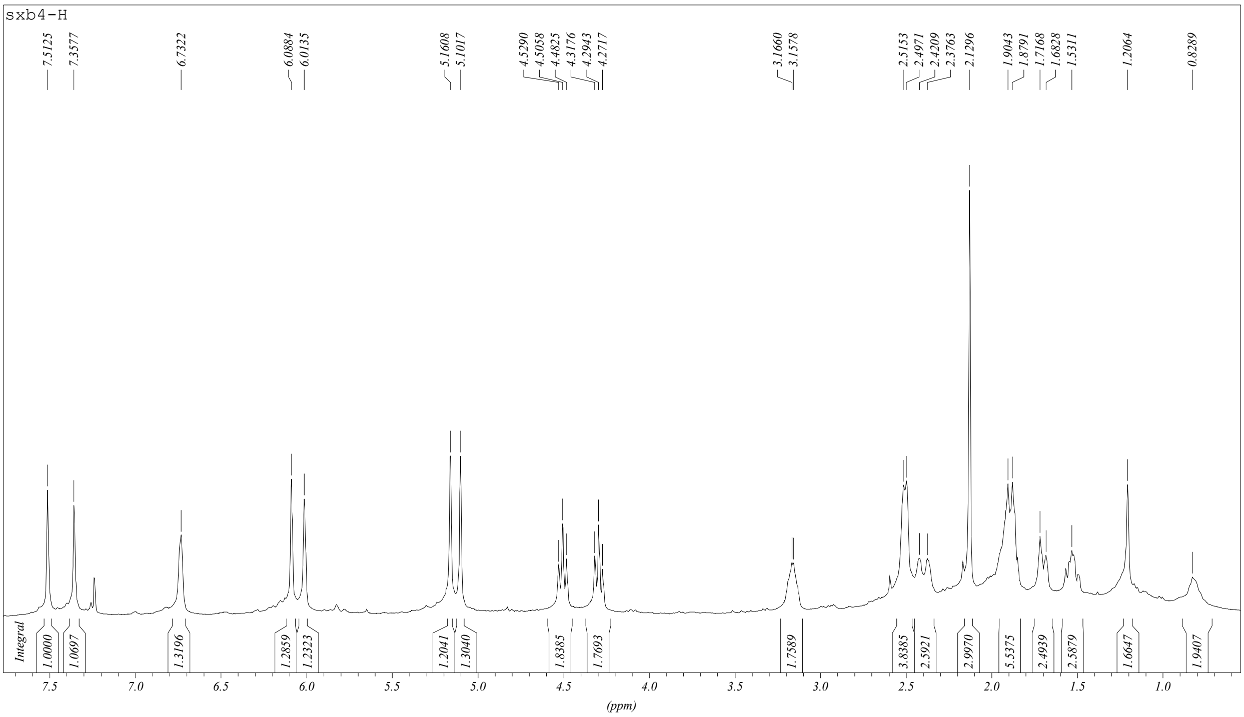

Spectra 2. ^{1}H NMR spectrum of **1**

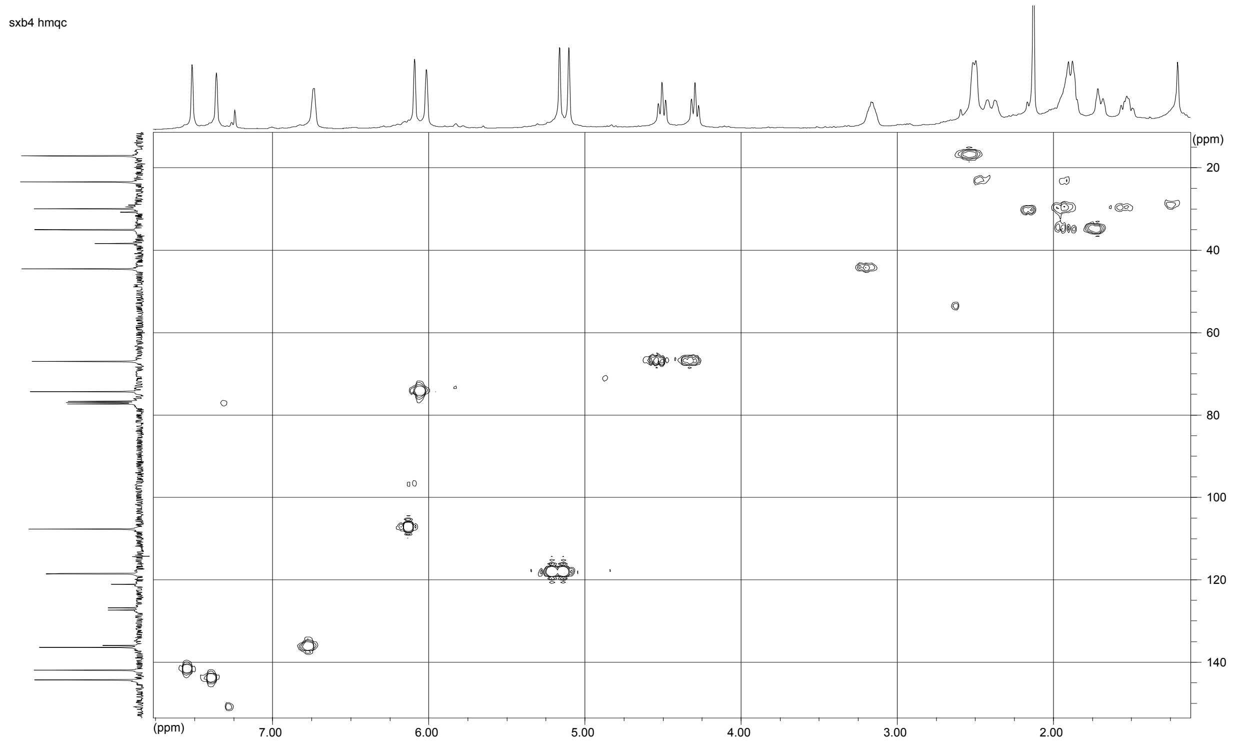

Spectra 3. HSQC spectrum of **1**

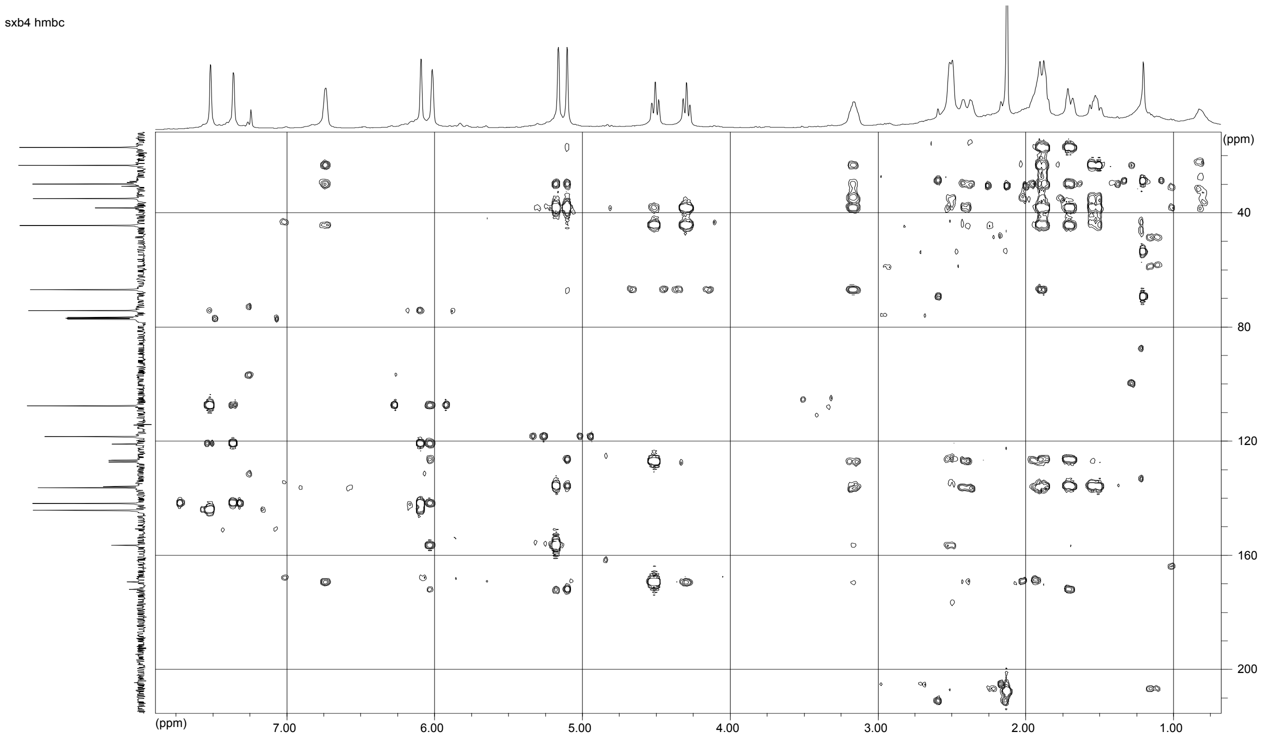

Spectra 4. HMBC spectrum of **1**

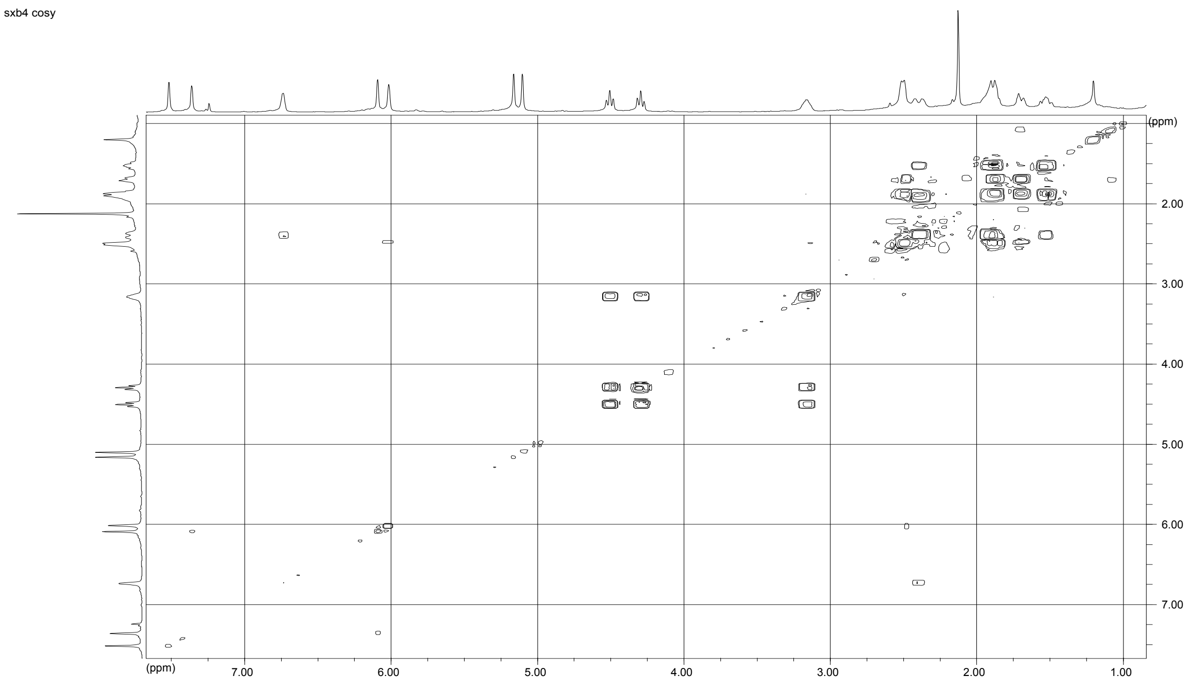

Spectra 5. COSY spetrum of **1**

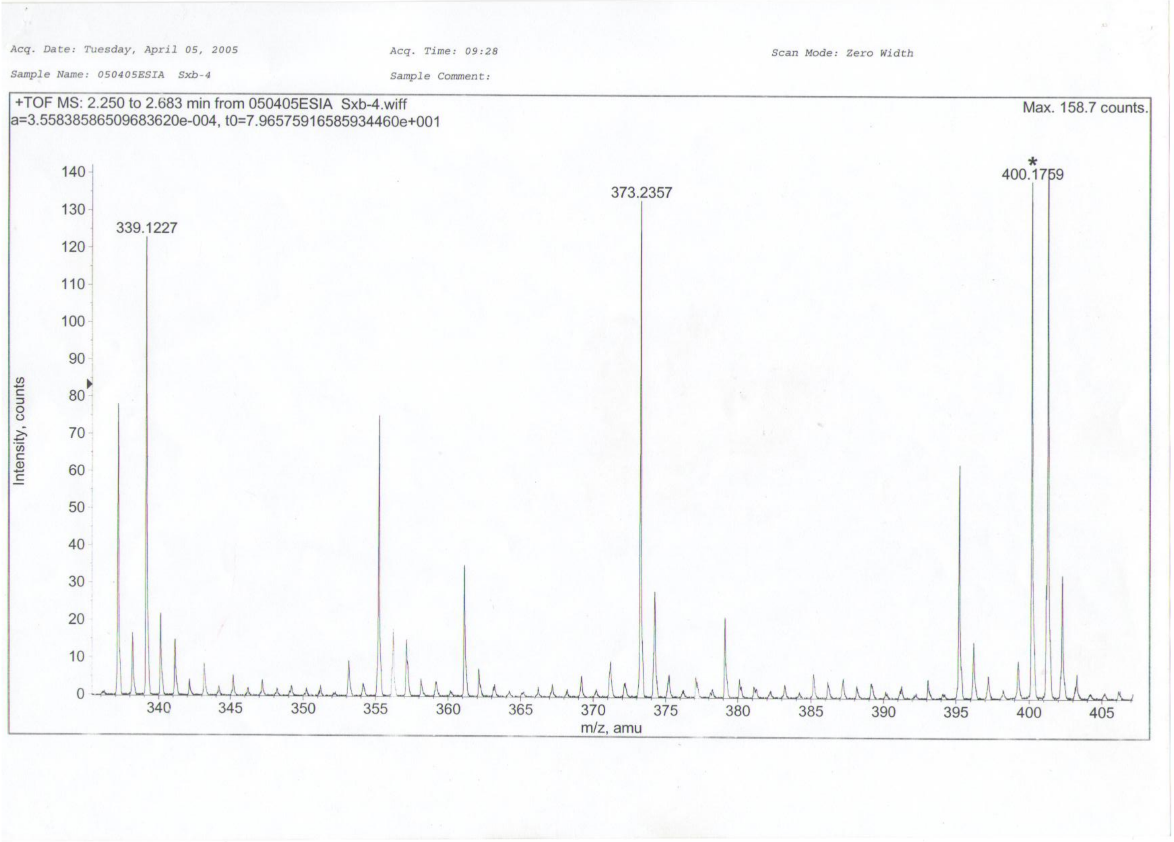

Spectra 6. HRESIMS data of **1**

For compounds 2:

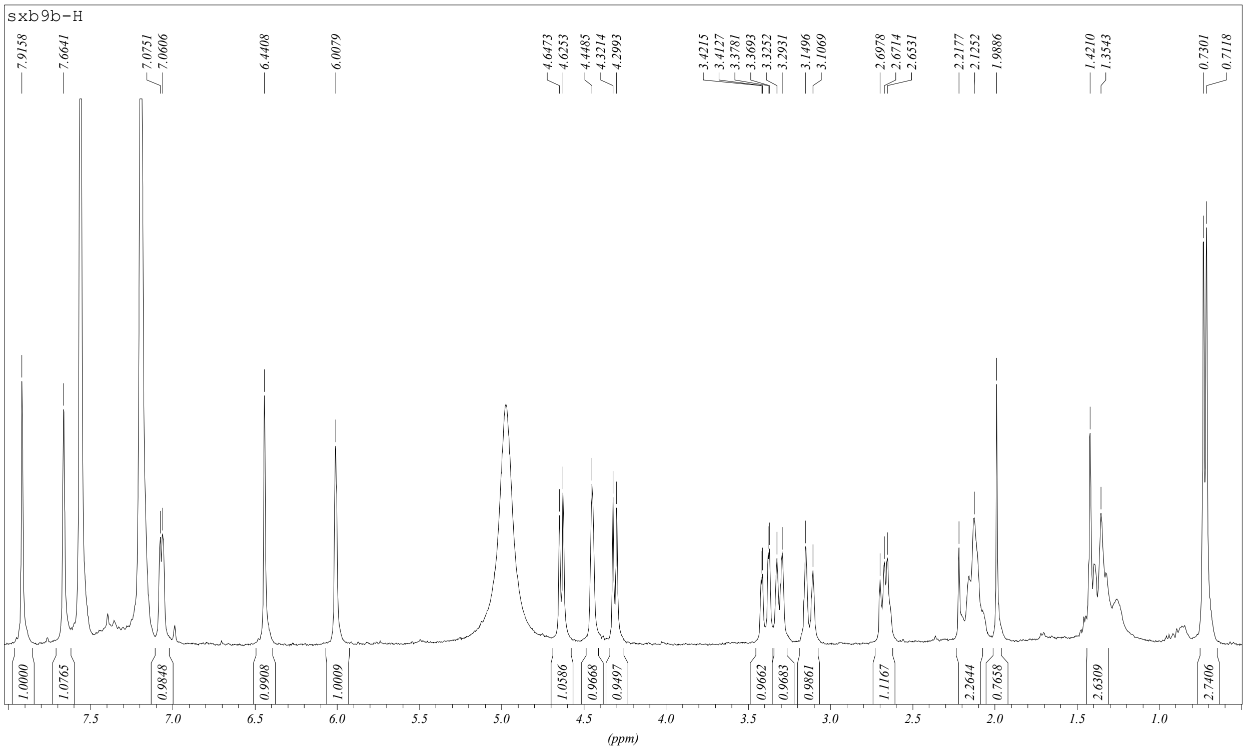

Spectra 7. ^{1}H NMR spectrum of **2**

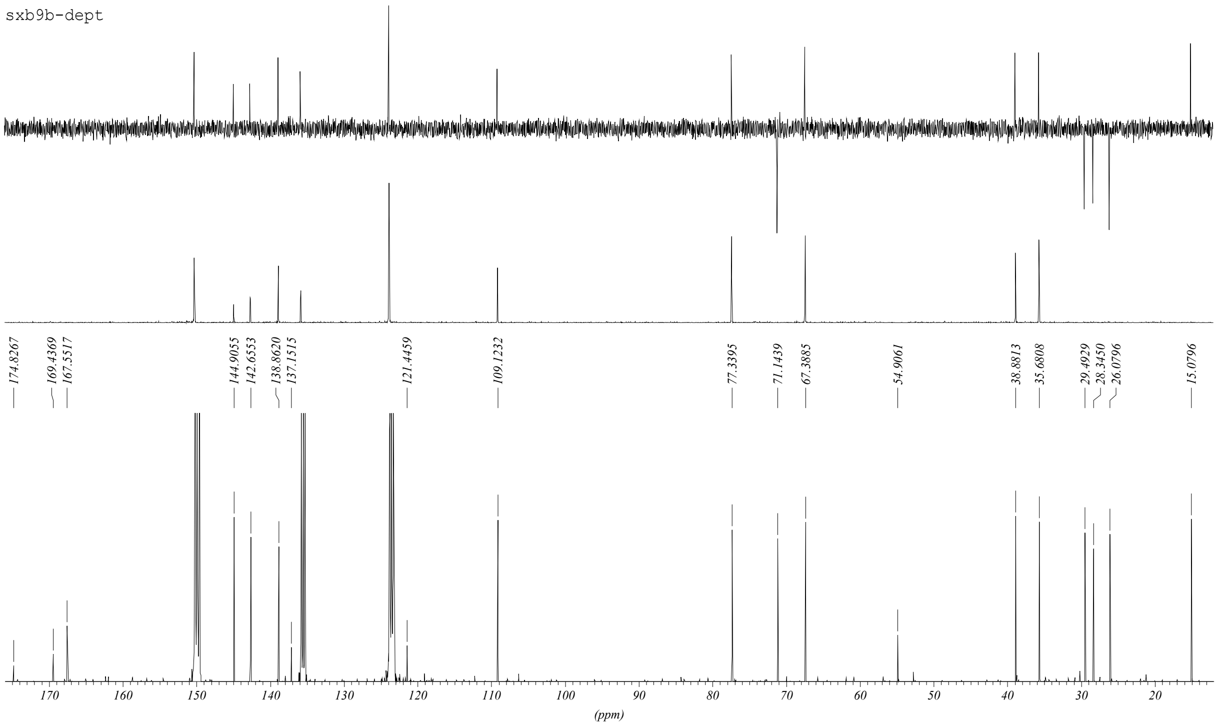

Spectra 8. ^{13}C NMR and DEPT spectra of **2**

For compounds 3:

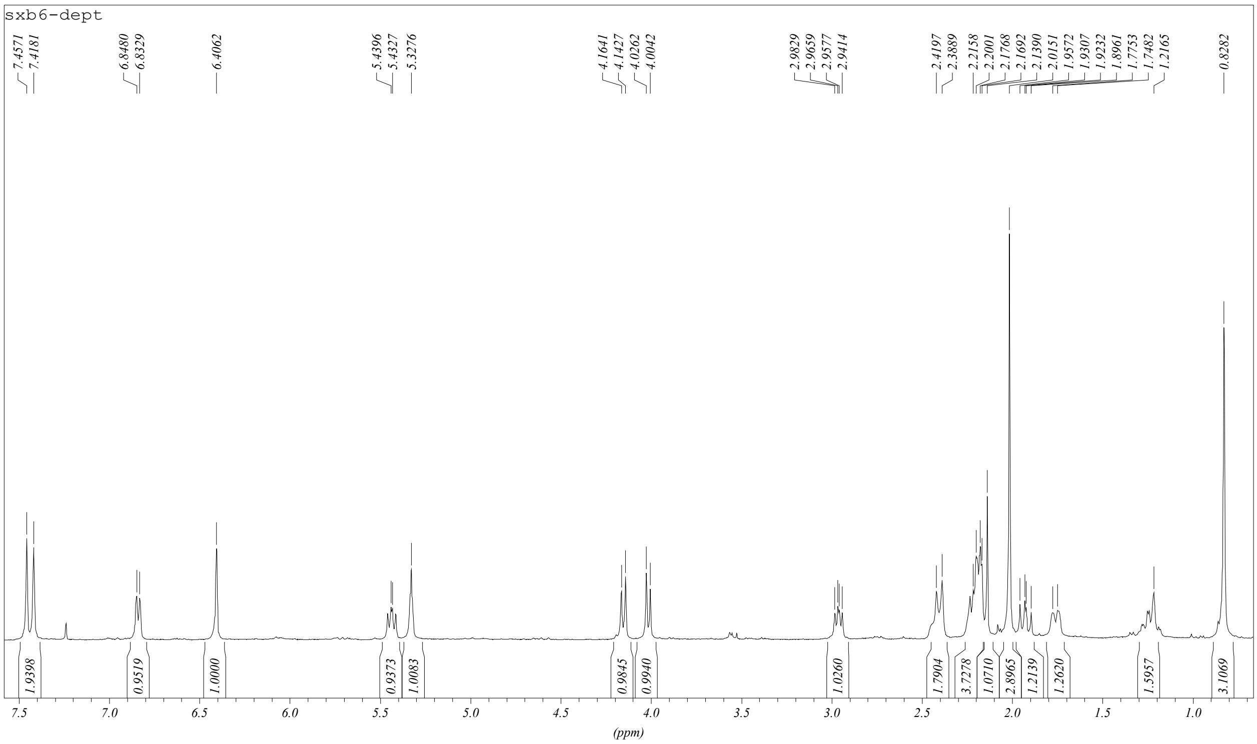

Spectra 9. ^{1}H NMR spectrum of **3**

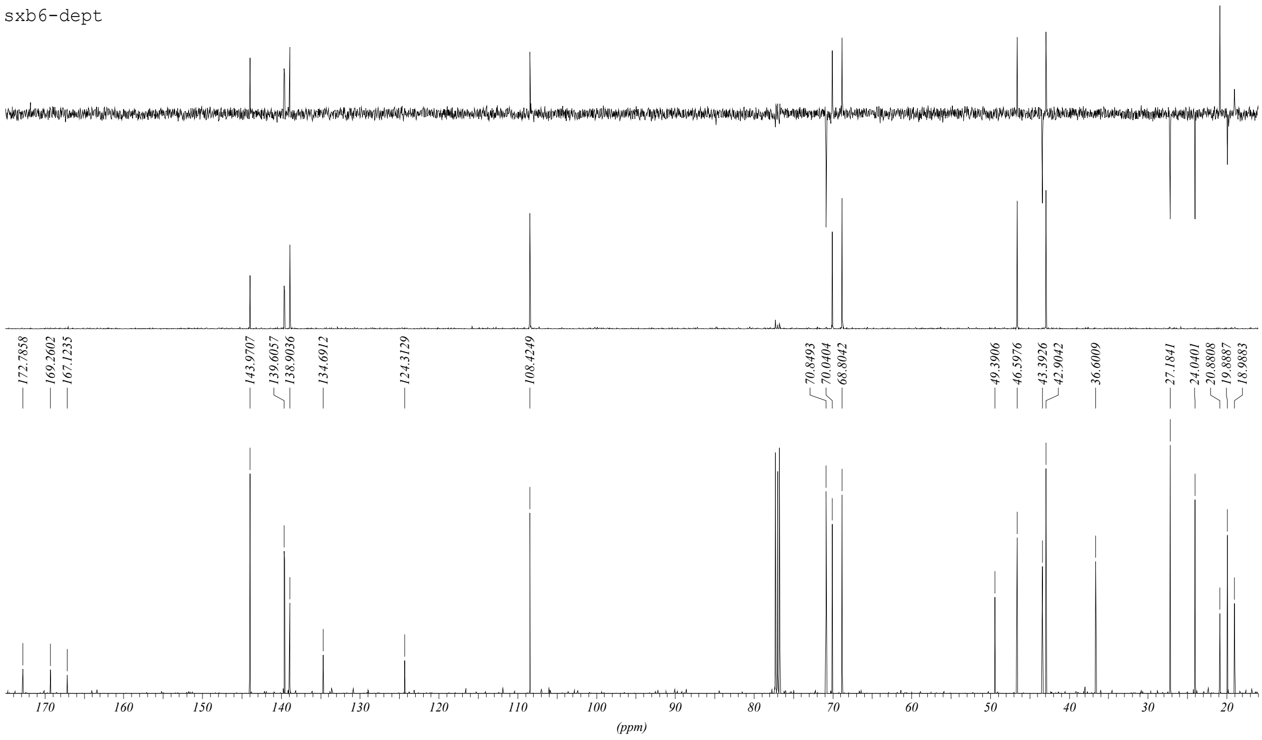

Spectra 10. ^{13}C NMR and DEPT spectra of **3**

For compound 4:

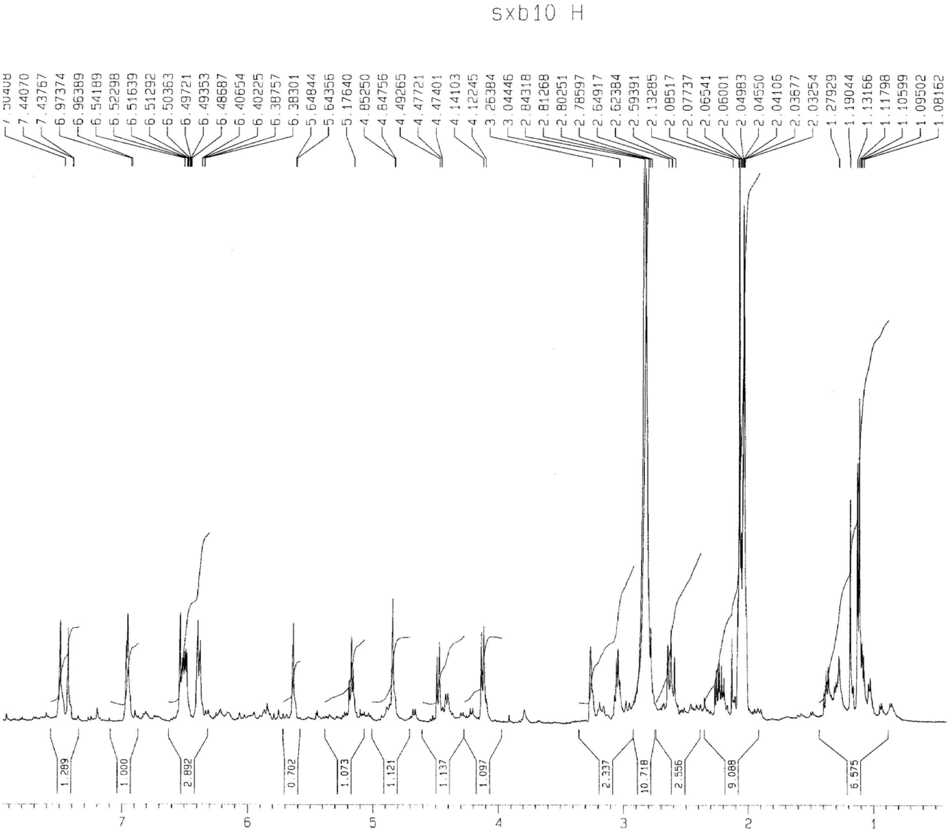

Spectra 11. ^{1}H NMR spectrum of **4**

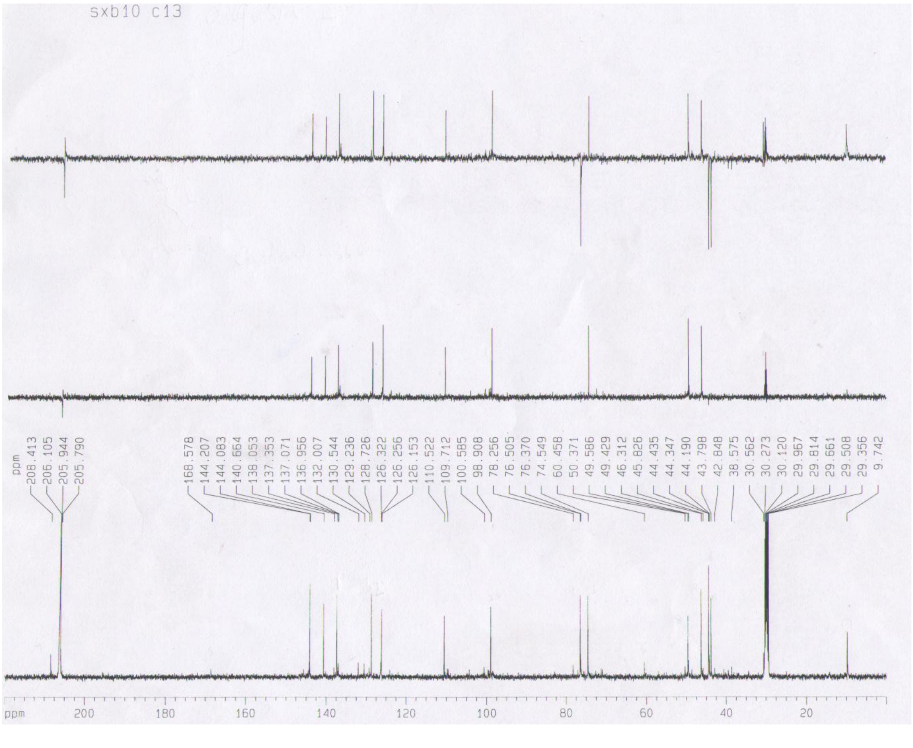

Spectra 12. ^{13}C NMR and DEPT spectra of **4**

For compound 5:

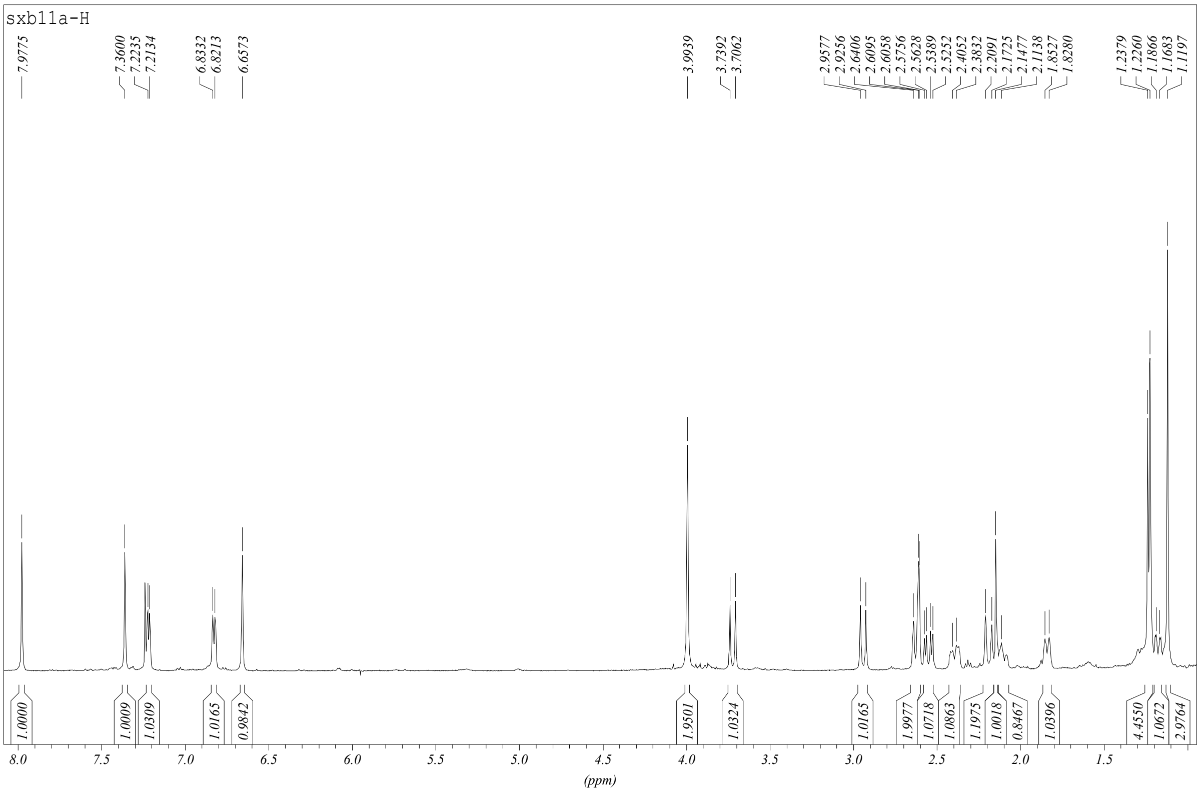

Spectra 13. ^{1}H NMR spectrum of **5**

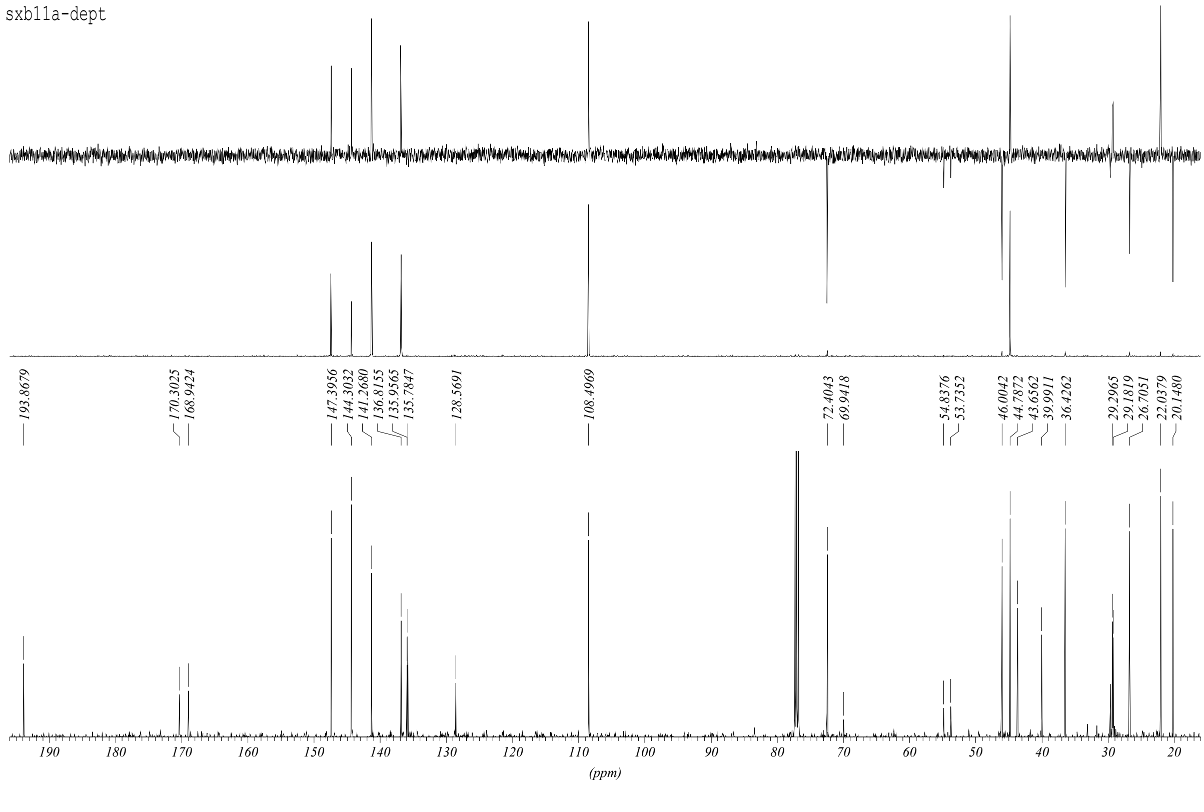

Spectra 14. ^{13}C NMR and DEPT spectra of **5**
